# Serum Asprosin Level as a New Biomarker in Differentiating Familial Mediterranean Fever Attacks

**DOI:** 10.7759/cureus.35342

**Published:** 2023-02-23

**Authors:** Hilal Sipahioglu, Ozlem Sen, Sümeyra Koyuncu, Sibel Kuzugüden

**Affiliations:** 1 Department of Internal Medicine, Kayseri City Training and Research Hospital, Kayseri, TUR; 2 Department of Clinical Biochemistry, Kayseri City Education and Research Hospital, Kayseri, TUR

**Keywords:** free atack, anti-inflammatory, attack, familial mediterranean fever, asprosin

## Abstract

Introduction

Familial Mediterranean fever (FMF) is a recessively inherited disease characterized by recurrent attacks of fever and sterile polyserositis. Recently, some proteins originating from adipose tissue have been demonstrated to play a critical role in the inflammatory process. Asprosin is a new adipokine secreted by adipose tissue, and proinflammatory cytokines have been determined to increase with the decrease of circulating asprosin. This study was designed to evaluate the level of asprosin in the acute attack and attack-free period in FMF patients.

Materials and methods

A total of 65 FMF patients were evaluated for this cross-sectional case-control study. Those who were obese and had concomitant diabetes mellitus, hypertension, heart failure, and rheumatological disease were excluded from the study. The patients were divided into two groups: attack-free period and attack period. Fifteen healthy individuals who were not obese and had no additional disease were included as the control group. Demographic data, gene analyses, laboratory findings, and symptoms were recorded at the time of diagnosis. Serum asprosin level was studied by enzyme-linked immunosorbent assay test in the outpatient clinic controls of the patients. Asprosin levels and other laboratory findings were compared between the attack, attack-free, and control groups.

Results

Of the patients included in the study, 50% were in the attack period, and 50% were in the free-attack period. The mean age of the FMF patients was 34±10 years. Asprosin level in the control [median (interquartile range (IQR))=30.4 (21.5-57.7) ng/mL] group was significantly higher than the attack [median (IQR)=21.5 (17.5-28) ng/mL] and attack-free [median (IQR)=19(18.7-23) ng/mL] groups (p=0.001). C-reactive protein and sedimentation levels were significantly higher in the attack group compared to the other two groups (p<0.001).

There was a moderate correlation between C-reactive protein and asprosin levels (Ro=-0.314, p=0.01). The cut-off value of serum asprosin level was determined as 21.6 ng/mL; sensitivity was 78%, and specificity was 77% (p<0.001).

Conclusion

The study demonstrated that the serum asprosin levels of FMF patients with acute attack were lower than those in the attack-free periods and healthy controls. Asprosin is likely to have a role in the anti-inflammatory cascade.

## Introduction

Familial Mediterranean fever (FMF) is a systemic familial disease characterized by inflammatory reactions of the serosal membranes and recurrent episodes of abdominal, chest, and joint pain, skin rashes, and fever [1]. During attack periods, several metabolic and immunological changes occur in FMF patients. The subclinical inflammation may persist in the period between attacks, and increased release of proinflammatory cytokines (including interleukin-6, interleukin-8, and tumor necrosis factor-α [TNF-α]), selectins (P, E, and L-selectins), and peptides (e.g., serotonin and thrombomodulin) may play a role in FMF’s inflammatory phases [2]. In recent years, a series of bioactive substances called adipokines secreted from adipose tissue has been demonstrated to contribute towards the complications of obesity through the regulation of inflammatory and immune responses [3,4]. Adipocytes produce several adipocytokines, growth factors, and hormones affecting metabolic processes and endothelial function [5]. Adipokines also include proinflammatory cytokines such as leptin, TNF-α, and IL-6 [6]. Asprosin, a novel adipokine secreted by adipose tissue, peaks during fasting and induces hepatic glucose release through the activation of the G protein-cAMP-PKA pathway, which has been indicated to have a curative effect on chronic inflammation [7,8]. Decreased circulating asprosin in rats with hypomorphic fibrillin-1 allele caused the impairment of glucose homeostasis and the elevation of proinflammatory cytokines and contributed to the formation of inflammatory diseases such as aortic aneurysms [9,10]. These are findings that may highlight possible anti-inflammatory aspects of asprosin. Asprosin is encoded by the fibrillin-1 gene, which also encodes the protein fibrillin-1. Fibrillin-1 insufficiency has been shown to interfere with wound healing in connective tissue damaged by inflammatory diseases [11].

This study was designed to evaluate the level of asprosin, whose anti-inflammatory effect has been demonstrated previously, in acute attack and attack-free periods in FMF patients.

## Materials and methods

Patients over the age of 18 who applied to the rheumatology outpatient clinic between March and September 2022 with FMF diagnoses were included in the study. Kayseri City Hospital Ethics Committee issued approval 587/ 24.02.2022. Patients with a body mass index >25 in men and >30% in women with diabetes mellitus, heart failure, other rheumatological diseases, and hypertension were excluded. The control group consisted of healthcare professionals working in our hospital.

Demographic data of the patients, the time of the diagnosis, symptoms at the time of diagnosis, FMF gene mutation status, presence of splenomegaly, and amyloidosis were recorded from the patient files. Along with the routine examinations when the patients applied to the outpatient clinic, a blood sample was taken from the patient into a 4 cc biochemistry tube after the patient's consent was obtained. After the collected blood was centrifuged at 3000 rpm for five minutes, 3 ml of serum supernatant was removed and collected in an Eppendorf tube. Serum samples were kept frozen at −80°C. Serum asprosin protein concentrations were analyzed using the enzyme-linked immunosorbent assay method (Cat.No: E4095HU; BT Lab, Shanghai, China). The patients included in the study were diagnosed with FMF according to Tel-Hashomer criteria, and then the patients were divided into attack and attack-free groups [2]. Laboratory values and serum asprosin levels in attack, attack-free, and control groups were evaluated in FMF patients.

Statistical analysis

Statistical analysis was performed using the IBM SPSS statistics program version 22 (IBM Corp., Armonk, NY, USA). The normality distributions of continuous variables were examined using the Shapiro-Wilk test. According to the normal distribution, continuous variables were presented as mean±SD or median (interquartile range) values. Categorical variables were shown as numbers (%, percentage). The differences in numerical variables among different groups were analyzed by the ANOVA test. Chi-square test compared categorical variables and nominal variables.

The correlation between C-reactive protein (CRP) and asprosin level was investigated using the Spearman correlation test. The correlation coefficient was accepted as 0-0.29 (weak), 0.30-0.69 (moderate), and 0.70-1.0 (strong). A receiver operating characteristic (ROC) curve was constructed to determine the best cut-off values for serum asprosin for the attack in FMF. In all analyses, a p-value of <0.05 was considered statistically significant.

## Results

A total of 65 patients with a diagnosis of FMF were evaluated. Five patients were excluded from the study for having diabetes mellitus, three for hypertension, four for having other rheumatological diseases, and five due to excessive hemolysis of blood.

A total of 48 FMF patients were included; 24 (50%) were in the attack period, and 24 (50%) were in the attack-free period. The mean age of the FMF patients was 34±10 years, and the mean age of onset of symptoms was 21±7 years. All patients had serositis at the time of diagnosis. The demographic and clinical characteristics of the patients are presented in Table 1.

**Table 1:** Demographic and clinical characteristics of the patients

Variables	n=48(%)
Age (year)	34±10
Female n(%)	19(40)
Age at symptom onset (year)	21±7
Number of attacks within the last year	2(4)
Fever n(%)	46(96)
Amyloidosis n(%)	2(4)
Serositis n(%)	48(100)
Arthritis n(%)	5(10)
Skin rash n(%)	2(4)
M694V Homozygous gene mutation n(%)	15(31)
M694V Heterozygous gene mutation n(%)	15(31)
Other Exon 10 Heterozygous gene mutation n(%)	8(17)
Exon 2 Heterozygous gene mutation n(%)	2(4)
Compound heterozygous gene mutation n(%)	8(17)

BMI and laboratory findings of the patients in the attack, attack-free, and control groups are compared in Table 2. The BMIs of the patients and the healthy control group were similar (p>0.05). Asprosin level was significantly higher in the control group than in the attack and attack-free groups (p=0.001). CRP and sedimentation levels were significantly higher in the attack group compared to the other two groups (p<0.001).

**Table 2:** Biochemical laboratory values of control, attack-free, and attack groups when compared to the control BMI; Body Mass Index, CRP; C-reactive protein, ESR; erythrocyte sedimentation rate, HDL-C; high-density lipoprotein cholesterol, IQR; interquartile range, LDL-C; low-density lipoprotein cholesterol, TG; triglyceride; a, b and c; Different superscripts among groups indicate a statistically significant difference between groups. *p=0.001, ** p<0.001

Variables	Control n=15	Attack-free n=24	Attack n=24
BMI mean±SD	24(22-28)	26(23-28)	26(22-29)
Asprosin ng/mL median(IQR)^*^	30.4(21.5-57.7)^a^	21.5(17.5-28) ^b^	19(18.7-23) ^c^
CRP mg/L median(IQR)^**^	1.1(0.7-2)^a^	1(0.6-2.1)^a^	14(10-26)^b^
ESR mm/h median(IQR)	5(3-6)^a^	4(3-9)^a^	19.5(8.7-29) ^b^
Fibrinogen mean±SD^**^	2569±381^a^	2926±596^a^	3710±647^b^
Creatinine mg/dL mean±SD	0.6±0.1	0.7±0.1	0.69(0.54-0.79)
Hemoglobin g/dL mean±SD	13.8±1.7	15±1.7	13.2-2.2
White blood cell/mm3 mean±SD	7384±1720	6944±2765	7764±2019
Neutrophil/mm3 median(IQR)	4890(3390-5750)	3960(2810-4850)	4675(3317-5532)
Lymphocyte/mm3 mean±SD	2244±490	2570±1509	2308±698
Monocyte /mm3 mean±SD	524±114	543±224	501±154
HDL-C mg/dL mean±SD	47±12	42±10	43±9
LDL-C mg/dL mean±SD	113±33	105±35	104±34
TG mg/dL median(IQR)	94(78-148)	137(86-227)	95(79-164)

**Figure 1:**
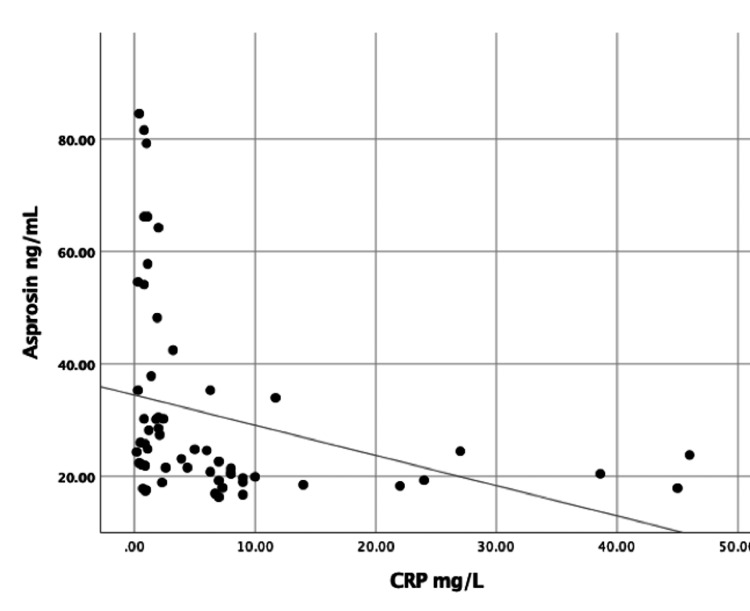
Correlation between asprosin and CRP in patients with FMF (Ro=-0.314 p=0.01) FMF; familial Mediterranean fever, CRP; C-reactive protein

**Figure 2:**
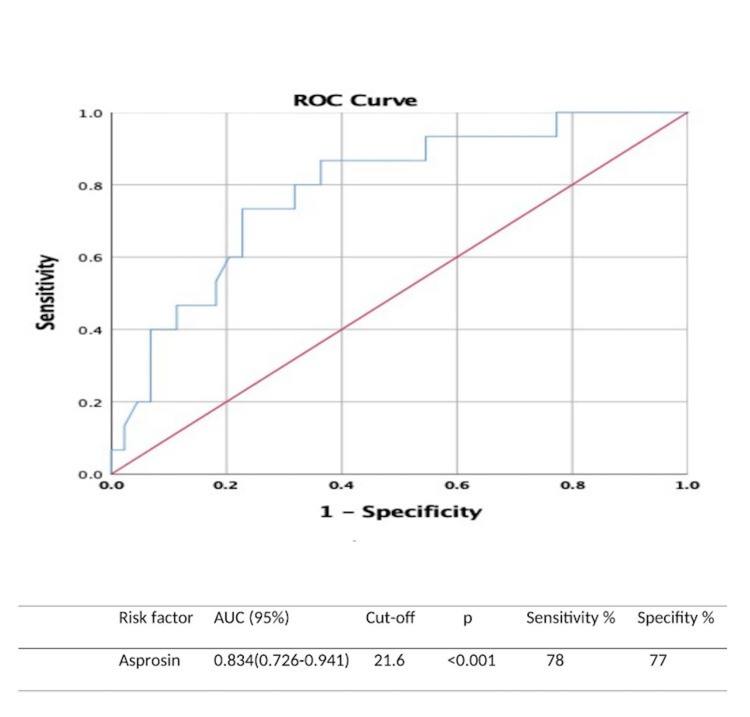
ROC curve of asprosin level for FMF attack FMF; familial Mediterranean fever, AUC; Area Under Curve, ROC curve; Receiver Operating Characteristic curve

Figure 1 presents a moderate correlation between CRP and asprosin levels (Ro=-0.314 p=0.01). The ROC curve for the FMF attack is shown in Figure 2. The cut-off value of serum asprosin level was 21.6 ng/mL, while the sensitivity was 78% and the specificity was 77% (p<0.001).

## Discussion

In our study, asprosin level was lower in patients in the attack and attack-free periods compared to the healthy population (p=0.001). At the same time, a moderately significant negative correlation was determined between CRP, a critical indicator for the attack period of FMF, and asprosin level (p=0.001).

Among the findings supporting the diagnosis in FMF patients, the increase in the blood levels of acute phase reactants during the attack is crucial, and excessive activation of cytokine cascades during the attack has been demonstrated [12,13]. Both in attack-free and attack periods, proinflammatory cytokines significantly increase. Abnormal pyrin protein, created as a result of MEFV gene mutations, neutralizes the blocking suppression of inflammation and is considered responsible for this ongoing inflammatory process [14,15]. Diagnostic testing is still needed to identify FMF and monitor inflammatory activity.

Asprosin, discovered in recent years as a protein-structured hormone that regulates hepatic glucose secretion, is the C-terminal cleavage product of the profibrillin-1 protein. This new adipokine hormone is secreted from white adipose tissue and increases glucose and insulin secretion during fasting [16]. Macrophages are the immune cell type that accumulates most in adipose tissue depots [17]. There are primarily two main phenotypes of macrophages: M1 (classic), which promotes inflammation, and M2 (alternative), which suppresses it. The transition between these phenotypes is regulated by signals from other immune cells and adipocytes [18,19]. It is not known exactly how asprosin has an effect on this pathway; therefore, our study can be a guide regarding this subject.

Wang et al., in a study with ulcerative colitis and Crohn's patients with active inflammation and attacks similar to FMF, determined higher asprosin levels in the control group, similar to our study. Furthermore, a significant increase in asprosin levels was observed after treatment in patients with ulcerative colitis and Crohn's disease [20]. The main purpose of treatment in FMF is to reduce the frequency and severity of attacks and prevent the development of amyloidosis. According to our study results, aiming to increase the level of asprosin may reduce the number and severity of attacks. Asprosin has also been demonstrated in some studies to contribute to wound healing, have protective effects on the myocardium, ameliorate endothelial damage, and significantly improve left ventricular functions [21,22].

Omentin, another adipokine such as asprosin, was reported to be at a significantly lower level in FMF patients compared to the control group, similar to our study. In fact, this adipokine was lower in patients taking colchicine [23]. In the study of Gerdan et al., adipokines (leptin and adiponectin) and ghrelin were significantly lower in the FMF patient population compared to the control group, and there was a negative correlation with CRP [24].

In COVID-19 disease, an increase in inflammation occurs due to the increase in cytokines. Seyhanlı et al. revealed that asprosin level was lower in patients with COVID-19 compared to the healthy control group, similar to our study [25].

In FMF patients, the cut-off value of the asprosin level was 21.6 ng/dL in showing the attack period, and the sensitivity and specificity were higher than 70%. We consider that asprosin level may be a good biomarker to evaluate inflammatory activity in FMF patients. 

The limitations of our study are the small number of patients, the inclusion of patients in a single center, and the inability to examine cytokine levels in patients due to low cost.

## Conclusions

In conclusion, to the best of our knowledge, this study is the first to examine asprosin levels in FMF patients. Asprosin level was lower in FMF patients compared to the healthy control group. Moreover, we demonstrated a negative correlation with the level of CRP, an indicator of inflammation in the attack period. However, studies with larger patient populations are needed to clarify its pathophysiology better.

## References

[1] Ozdogan H, Ugurlu S (2019). Familial Mediterranean fever. Presse Med.

[2] Alghamdi M (2017). Familial Mediterranean fever, review of the literature. Clin Rheumatol.

[3] Ouchi N, Kihara S, Funahashi T, Matsuzawa Y, Walsh K (2003). Obesity, adiponectin and vascular inflammatory disease. Curr Opin Lipidol.

[4] Berg AH, Scherer PE (2005). Adipose tissue, inflammation, and cardiovascular disease. Circ Res.

[5] Oh DK, Ciaraldi T, Henry RR (2007). Adiponectin in health and disease. Diabetes Obes Metab.

[6] Bevilacqua MP, Nelson RM (1993). Selectins. J Clin Invest.

[7] Romere C, Duerrschmid C, Bournat J (2016). Asprosin, a fasting-induced glucogenic protein hormone. Cell.

[8] Birrell MA, Maher SA, Dekkak B, Jones V, Wong S, Brook P, Belvisi MG (2015). Anti-inflammatory effects of PGE2 in the lung: role of the EP4 receptor subtype. Thorax.

[9] Ju X, Ijaz T, Sun H (2014). IL-6 regulates extracellular matrix remodeling associated with aortic dilation in a fibrillin-1 hypomorphic mgR/mgR mouse model of severe Marfan syndrome. J Am Heart Assoc.

[10] Wang Y, Qu H, Xiong X (2018). Plasma asprosin concentrations are increased in individuals with glucose dysregulation and correlated with insulin resistance and first-phase insulin secretion. Mediators Inflamm.

[11] Handa K, Abe S, Suresh VV (2018). Fibrillin-1 insufficiency alters periodontal wound healing failure in a mouse model of Marfan syndrome. Arch Oral Biol.

[12] Cekin N, Akyurek ME, Pinarbasi E, Ozen F (2017). MEFV mutations and their relation to major clinical symptoms of familial Mediterranean fever. Gene.

[13] Touitou I, Milhavet F, Cuisset L (2014). Response to Li and Zhang: infevers, a human gene mutation database for autoinflammatory diseases including disseminated superficial actinic porokeratosis. J Dermatol Sci.

[14] Direskeneli H, Ozdogan H, Korkmaz C, Akoglu T, Yazici H (1999). Serum soluble intercellular adhesion molecule 1 and interleukin 8 levels in familial Mediterranean fever. J Rheumatol.

[15] Moradian MM, Babikyan D, Banoian D, Hayrapetyan H, Manvelyan H, Avanesian N, Sarkisian T (2017). Comprehensive analysis of mutations in the MEFV gene reveal that the location and not the substitution type determines symptom severity in FMF. Mol Genet Genomic Med.

[16] Donma MM, Donma O (2018). Asprosin: Possible target in connection with ghrelin and cytokine network expression in the post-burn treatment. Med Hypotheses.

[17] Guzik TJ, Skiba DS, Touyz RM, Harrison DG (2017). The role of infiltrating immune cells in dysfunctional adipose tissue. Cardiovasc Res.

[18] Martinez FO, Gordon S (2014). The M1 and M2 paradigm of macrophage activation: time for reassessment. F1000Prime Rep.

[19] Mouton AJ, Li X, Hall ME, Hall JE (2020). Obesity, hypertension, and cardiac dysfunction: novel roles of immunometabolism in macrophage activation and inflammation. Circ Res.

[20] Wang HH, Luo WY, Lin M, Li XJ, Xiang GD, D Triganti S (2021). Plasma asprosin, CCDC80 and ANGPTL4 levels are associated with metabolic and cardiovascular risk in patients with inflammatory bowel disease. Physiol Res.

[21] Guven C, Kafadar H (2022). Evaluation of plasma asprosin concentration in patients with coronary artery disease. Braz J Cardiovasc Surg.

[22] Chen S, Wang X, Qiu CM (2019). [Study of the role and mechanism of asprosin/spartin pathway in cardiac microvascular endothelial injury induced by diabetes mellitus]. Sichuan Da Xue Xue Bao Yi Xue Ban.

[23] Can Sandikci S, Omma A, Yucel C, Omma T (2021). Is there a relationship between serum omentin level and acute phase response in patients with familial Mediterranean fever?. Clin Rheumatol.

[24] Gerdan V, Sari I, Kozacı D (2012). Down-regulation of adiponectin in patients with familial Mediterranean fever during attack-free period. Rheumatol Int.

[25] Seyhanli ES, Koyuncu I, Yasak IH, Demir HA, Temiz E (2022). Asprosin and oxidative stress level in COVID-19 patients. Clin Lab.

